# Targeting Gut Microbiota With Natural Polysaccharides: Effective Interventions Against High-Fat Diet-Induced Metabolic Diseases

**DOI:** 10.3389/fmicb.2022.859206

**Published:** 2022-03-15

**Authors:** Chao-Yue Sun, Zuo-Liang Zheng, Cun-Wu Chen, Bao-Wei Lu, Dong Liu

**Affiliations:** ^1^College of Biological and Pharmaceutical Engineering, West Anhui University, Lu’an, China; ^2^School of Life Science, Jiaying University, Meizhou, China

**Keywords:** high-fat diet, metabolic diseases, gut microbiota, polysaccharides, obesity

## Abstract

Unhealthy diet, in particular high-fat diet (HFD) intake, can cause the development of several metabolic disorders, including obesity, hyperlipidemia, type 2 diabetes mellitus (T2DM), non-alcoholic fatty liver disease (NAFLD), and metabolic syndrome (MetS). These popular metabolic diseases reduce the quality of life, and induce premature death worldwide. Evidence is accumulating that the gut microbiota is inextricably associated with HFD-induced metabolic disorders, and dietary intervention of gut microbiota is an effective therapeutic strategy for these metabolic dysfunctions. Polysaccharides are polymeric carbohydrate macromolecules and sources of fermentable dietary fiber that exhibit biological activities in the prevention and treatment of HFD-induced metabolic diseases. Of note, natural polysaccharides are among the most potent modulators of the gut microbiota composition. However, the prebiotics-like effects of polysaccharides in treating HFD-induced metabolic diseases remain elusive. In this review, we introduce the critical role of gut microbiota human health and HFD-induced metabolic disorders. Importantly, we review current knowledge about the role of natural polysaccharides in improving HFD-induced metabolic diseases by regulating gut microbiota.

## Introduction

Diet can affect multiple aspects of human health, and high-fat diet (HFD) contributes to the epidemics of obesity and obesity-associated chronic metabolic diseases, such as diabetes, hyperlipidemia, hypertension, non-alcoholic fatty liver disease (NAFLD), and metabolic syndrome (MetS; Li et al., 2016). These metabolic diseases remain a serious global burden that kill at least 2.5 million people annually (Li et al., 2016). Interestingly, metabolic diseases patients are highly associated with increased risk of developing the severe coronavirus disease 2019 (COVID-19) (Le Roux, 2021; Steenblock et al., 2021). In addition, over-consumption of high-fat directly promotes the tumorigenesis, such as prostate cancer, colorectal cancer (CRC), and hepatocellular carcinoma (Beyaz et al., 2016; Labbe et al., 2019; Yang et al., 2022).

Gut microbiota is considered as an invisible “forgotten organ” of human beings that is composed of trillions of microorganisms (Ursell et al., 2012). As a real organ, it is host-specific, and affects almost all aspects of host physiology, which can be effectively modified by diet, or surgery (Marchesi et al., 2016). Due to the development of next-generation sequencing technologies, the genomic blueprint of human gut microbiota is visualized, and our understanding the functions of intestinal microbiota in human health becomes more accurate (Almeida et al., 2019). The gut microbiota plays important role in nearly all aspects of human health and disease. For example, alterations of intestinal microbiota are highly correlated with levels of SARS-CoV-2, and severity of patients with COVID-19 (Zuo et al., 2020). In addition, maternal gut microbiota also influences the metabolic phenotype and social behavior of offspring (Buffington et al., 2016; Kimura et al., 2020; Laursen et al., 2021). Notably, in a phase 3, double-blind and randomized trial, oral administration of gut microbiome-derived drug (SER-109) reduces the risk of recurrent infection of *Clostridioides difficile* infection (Feuerstadt et al., 2022).

The intestinal microbiota composition is strongly associated with human diseases, especially chronic metabolic disorders, such as obesity and type 2 diabetes mellitus (T2DM; Gentile and Weir, 2018). Gut microbiota dysbiosis, including reduced diversity, stability, and increased adverse bacteria, is frequently observed in patients with metabolic diseases, and gut microbiota-targeting intervention is an effective treatment for these diseases (Tilg and Moschen, 2014; Liu et al., 2021b). Alterations of intestinal microbiota are highly associated with tumorigenesis, especially CRC, and the intestinal microbiota have the potential as CRC biomarkers (Wong and Yu, 2019). The phenotypes of intestinal microbiota are distinct stage-specific in CRC patients, and the metabolites of the entire gut microbiota substantially influence the progression of CRC (Yachida et al., 2019). Accordingly, administration of probiotic, *Lactobacillus gallinarum* that is depleted in the intestine of CRC patients, protects against CRC tumorigenesis through producing protective metabolites (Sugimura et al., 2021). In addition, the gut microbiota affects human immune system, and the link between gut microbiota and the immune system is based on peripheral white blood cell (WBC) dynamics (Schluter et al., 2020). In cancer immunotherapy, the intestinal microbiota influences the toxicity of combined immune checkpoint blockade treatment (Andrews et al., 2021). Accordingly, diet intervention can impact human immune status by modulating “diet-microbiota-immune” axis, and high fermented food diet, for example, increases gut microbiome diversity and reduces inflammation (Wastyk et al., 2021). Moreover, the gut microbiota substantially affects the nervous system disorders through the “gut-brain” axis (Aaldijk and Vermeiren, 2022). For example, germ-free mice exhibit autism spectrum disorder (ASD)-like behaviors when harbored with intestinal microbiota from ASD patients, and the abnormalities of social behavioral can be improved by the administration of specific metabolites (Sharon et al., 2019).

Natural polysaccharides, one of the most the abundant dietary components, are polymeric carbohydrate macromolecules and sources of fermentable dietary fiber that exhibit biological activities with low toxicity, such as, anti-oxidation, anti-inflammatory, anti-tumor, and antiviral (Chaisuwan et al., 2021; Li et al., 2021a; Mirzadeh et al., 2021). Of note, natural polysaccharides are among the most potent modulators of the gut microbiota composition and benefit for human health (Yin et al., 2020; Song et al., 2021). However, the prebiotics-like activities of polysaccharides in treating HFD-induced metabolic diseases remain elusive. In this review, we introduce the critical role of gut microbiota human health and HFD-induced metabolic disorders. Notably, we focus on the role of natural polysaccharides in treating HFD-induced metabolic diseases by the “polysaccharides-gut microbiota” manner.

## High-Fat Diet Induces Gut Microbiota Dysbiosis

Increased HFD substantially alters intestinal microbiota composition and metabolites. Mice fed with HFD for short-term (2 days) exhibit altered gut microbiota composition at the phylum level, and longer-time HFD feeding (12 days) induces the gut microbiota dysbiosis (Ke et al., 2021). Regardless, after 2 or 12 days HFD feeding, the abundance of beneficial bacteria, *Bacteroidetes* is reduced, while the abundance of adverse bacteria, *Firmicutes* is substantially increased (Ke et al., 2021). The *Firmicutes*/*Bacteroidetes* ratio is increased in HFD-fed animals and humans and is proposed as a hallmark of obesity (Magne et al., 2020). In addition, the abundances of *Firmicutes* genus, including *Blautia*, *Bilophila*, *Enterorhabdus*, *Erysipelatoclostridium*, *Lactobacillus*, and *Oscillibacter*, is increased by HFD feeding, and these bacteria positively correlates with chronic metabolic diseases, such as obesity (Jo et al., 2021). Moreover, in zebrafish model, HFD feeding for 6 h increases intestinal microbiota abundance by 20 folds, and causes adverse effects in the bacterial communities (Ye et al., 2019). Importantly, germ-free mice prevent HFD-induced the insulin resistance, and increased cholesterol biosynthesis, suggesting that gut microbiota is involved in HFD-induced metabolic abnormalities (Rabot et al., 2010). In a randomized controlled trial, healthy young adults with high-fat consumption show adverse changes in gut microbiota, including increased *Alistipes* and *Bacteroides*, and decreased *Faecalibacterium* (Wan et al., 2019). In addition, HFD alters the functional integrity of the ileum, and one mechanism by which HFD induces gut microbes dysbiosis by modifying peroxisome proliferator-activated receptor-γ (PPAR-γ) pathway (Tomas et al., 2016). Of note, HFD-induced gut microbiome alterations are strongly associated with metabolic diseases (Kang et al., 2022). Currently, intervention of gut microbiota by prebiotics can prevent or ameliorate HFD-induced metabolic diseases (Kang et al., 2022). Of note, characterization of intestinal microbiota profiles prior to dietary intervention by prebiotics will increase the positive outcome in patients with metabolic disorders (Rodriguez et al., 2020). Thus, over-consumption of high-fat induces gut microbiota dysbiosis, and manipulation of gut microbiota can reverse HFD-induced metabolic diseases.

## Supplementation of Natural Polysaccharides Ameliorate HFD-Induced Metabolic Disorders by Modulating Gut Microbiota

Carbohydrates and polysaccharides are important composition of our daily diet that are not directly digested by the gastrointestinal enzymes (Shang et al., 2018). Carbohydrate fermentation is a critical function of the human gut microbiota, and then the fermented carbohydrates produce important metabolites in gut, such as short-chain fatty acids (SCFAs), and succinate (Canfora et al., 2019). Mechanistically, the gut microbiome harbors distinct enzymatic systems, including a glycoside hydrolase family of β-galactosidases and a carbohydrate-binding module family, to degrade plant-derived polysaccharides (Cabral et al., 2022). These processes are essential to maintain gut microbiota that depends mostly on non-digestively polysaccharides and fibers as energy sources (Sonnenburg and Sonnenburg, 2014). In turn, notably, polysaccharides are primary modulators of the function and composition of gut microbiota (Sonnenburg and Backhed, 2016; Yang et al., 2021a). Importantly, accumulating evidence demonstrates that supplementation of natural polysaccharides is an effective gut-microbiota-targeted treatment for HFD-induced metabolic disorders (Wang et al., 2018a; Su et al., 2021).

## HFD-Induced Obesity

The prevalence of obesity in children and adults, continues to increase and has tripled since 1975, and the rate of obese or overweight adults will account for 57.8% of global population by 2030 (Kelly et al., 2008; Loos and Yeo, 2021). Obesity is a serious public health concern that can induce premature death, and it is associated with increased risk of development of metabolic diseases, including diabetes, hypertension, hyperlipidemia, cardiovascular disease, and NAFLD. Obesity is a typical diet-related illness, and excess food uptake, especially HFD, is the most important master in the development of obesity (Kahn et al., 2006; Miyamoto et al., 2019). Increasing evidences demonstrate that HFD-induced obesity can cause adverse changes in intestinal microbial composition (Dalby et al., 2017; Miyamoto et al., 2019). Notably, obese individuals show increased the adverse bacteria, *Firmicutes*, *Fusobacteria*, *Proteobacteria*, and *Lactobacillus*, and decreased the beneficial bacteria, *Bacteroidetes*, *Verrucomicrobia*, *Faecalibacterium*, and *Lactobacillus plantarum* (Crovesy et al., 2020). One of the direct evidences for the role of gut microbiota in obesity is that, germ-free mice protects against obesity, despite the high-calories food intake (Bäckhed et al., 2004; Torres-Fuentes et al., 2017). In addition, germ-free mice colonized by intestinal microbiota from obese donors, display more increase of total body fat than lean donors (Turnbaugh et al., 2006). Mechanistically, gut bacteria and their metabolites regulate obesity through the “microbiota-gut-brain” axis (Torres-Fuentes et al., 2017; Cryan et al., 2019). Since intestinal microbiota dysbiosis can cause diet-related obesity, dietary intervention of gut microbiota is a therapeutic strategy of obesity (Zhang et al., 2015).

Natural polysaccharides from herbs and foods are important players in regulating gut microbiota to prevent or treat obesity (Lyu et al., 2017). Natural polysaccharides extracted from the *Artemisia sphaerocephala* Krasch seeds, alleviate HFD-induced obesity in mice by preventing HFD-induced gut microbiota dysbiosis, notably by reducing *Proteobacteria*, *AF12*, and *Helicobacter* (Li et al., 2020b). *Dictyophora indusiata* polysaccharides (DIP) shows anti-obesity effect in HFD-fed mice by reversing HFD-induced gut microbiota dysbiosis, notably by decreasing the ratio of bacteria, *Firmicutes*/*Bacteroidetes* (Kanwal et al., 2020). Microalgae polysaccharides isolated from *Chlorella pyrenoidosa*, *Spirulina platensis*, ameliorate obesity in HFD-fed mice by increasing the beneficial gut bacteria, including *Clostridia*, *Bacterioidia*, and *Mollicutes*, and reducing the unfavorable bacteria, *Actinobacteria* and *Verrucomicrobia* (Guo et al., 2021a). Modified apple polysaccharides (MAP) suppress HFD-induced obesity in mice by restoring HFD-induced gut microbiota disorder, notably by enriching the beneficial bacteria, *Bacteroidetes*, *Bacteroides*, and *Lactobacillus*, and inhibiting the adverse bacteria, *Fusobacterium* (Li et al., 2020d). Natural polysaccharides derived from *Raphanus sativus* display anti-obesity property in HDF-fed mice, and by reversing HFD-induced intestinal microbial dysbiosis *via* reducing the ratio of *Firmicutes*/*Bacteroidetes*, and increasing *Verrucomicrobia* (Do et al., 2021). Natural polysaccharides derived from WuGuChong ameliorate HDF-induced obesity in mice through decreasing the ratio of gut microbiota, *Firmicutes*/*Bacteroidetes* and the abundance of *Proteobacteria* (Wang et al., 2020). Polysaccharides derived from *Hirsutella sinensis mycelium* show anti-obesogenic effect in HFD-fed mice by modulating the gut microbiota composition, especially by enriching the gut bacteria, *Parabacteroides goldsteinii*. Importantly, oral administration of *P. goldsteinii* reduces obesity in HFD-fed mice (Wu et al., 2019b). Polysaccharides obtained from *Momordica charantia*, ameliorate HFD-induced obesity in mice by increasing the beneficial bacteria, such as *Actinobacteria*, *Coprococcus*, and *Lactobacillus*, and reducing the harmful bacteria, *Proteobacteria* and *Helicobacter* (Wen et al., 2021). Natural polysaccharides extracted from the food *Laminaria japonica*, alleviate HFD-induced obesity in mice by normalizing intestinal microbiota, notably by increasing the abundances of *Bacteroidales* and *Rikenellaceae* (Duan et al., 2019).

Intestinal microbial metabolites, particularly SCFAs, play crucial roles in the communication between gut microbes and the host (van de Wouw et al., 2018). Acetate, propionate, and butyrate are the most abundant SCFAs (>95%) in human body, and they are rapidly absorbed by the colonocytes (Dalile et al., 2019; He et al., 2020). The beneficial effects of SCFAs in obesity have been demonstrated, and SCFAs can protect against HFD-induced obesity (Den Besten et al., 2015). Notably, natural polysaccharides can promote intestinal SCFAs productions to ameliorate HFD-induced obesity. Alginate is the most abundant natural polysaccharides in *brown seaweed* that improves HFD-induced obesity in mice by increasing the SCFAs production, and gut bacteria, *Bacteroidales*, and reducing Clostridiales (Zheng et al., 2021). Mushroom polysaccharides isolated from *Pleurotus eryngii* display anti-obesity in HFD-fed mice through increasing the SCFAs-producing gut bacteria, *Anaerostipes* and *Clostridium* (Nakahara et al., 2020). Dietary *Enteromorpha clathrata* polysaccharides (ECP) attenuates obesity in HFD-fed mice by increasing the abundance of SCFAs, in particular, butyrate-producing gut bacterium, *Eubacterium xylanophilum* (Wei et al., 2021). *Lycium barbarum* polysaccharides (LBP) prevent obesity in HFD-fed mice, increase SCFAs, butyric acid and SCFAs-producing gut bacteria, *Lacticigenium*, *Lachnospiraceae*, and *Butyricicoccus* (Yang et al., 2021b). *Polygonatum odoratum* Polysaccharides attenuate HFD-induced obesity in rats by increasing the SCFAs, isobutyric acid, butyric acid, valeric acid, and reducing the adverse bacteria, *Actinobacteria*, *Proteobacteria*, and *Sutterella* abundance in intestine (Wang et al., 2018b). *Ganoderma lucidum* polysaccharides (GLP) inhibit HFD-fed obese mice by reversing HFD-induced gut microbiota disorder, and increasing SCFAs, acetate, and butyrate (Sang et al., 2021). Sulfated polysaccharides from *Stichopus japonicus*, prevents HFD-induced obesity in mice through enriching the probiotic *Akkermansia* and reducing the endotoxin-bearing *Proteobacteria*, and improving the SCFAs contents (Zhu et al., 2018). Mulberry leaf polysaccharides ameliorate HFD-induced obesity in mice by increasing the content of SCFAs, and reducing the *Firmicutes*/*Bacteroidetes* ratio (Li et al., 2021c). Taken together, natural polysaccharides exert prebiotics-like activities in preventing HFD-induced obesity by modulating the gut microbiota composition and metabolites.

## HFD-Induced Hyperlipidemia

Hyperlipidemia is an obesity-related metabolic disease characterized by high levels of total cholesterol, or triglyceride, or low-density lipoprotein (LDL) cholesterol. Hyperlipidemia is strongly associated with diet, and HFD accelerates *de novo* lipogenesis and increases the biogenesis of LDL (Yuan et al., 2019). Hyperlipidemia is an important risk factor for atherosclerotic cardiovascular diseases, metabolic disorders, and stroke (Soh et al., 2013). Recent studies show that gut microbiota is highly associated with hyperlipidemia and its related diseases, and gut microbiota can regulate lipid-metabolism homeostasis to develop hyperlipidemia (Jia et al., 2021). As such, manipulation of gut microbiota is an effective therapeutic option for hyperlipidemia (Kim et al., 2019). Currently, statins are major hypolipidemic drug, but with increased glioma risk (Cote et al., 2019). Natural polysaccharides can regulate gut microbiota to prevent or ameliorate HFD-induced hyperlipidemia.

Natural polysaccharides isolated from *Cordyceps militaris*, alleviate hyperglycemia in high-fat/sucrose diet-fed mice by improving gut microbiota dysbiosis, and promoting the abundance of probiotics, *Akkermansia muciniphila* (Lee et al., 2021). Natural polysaccharides isolated from seafood *Ostrea rivularis*, attenuate hyperlipidemia in HFD-fed zebrafish by improving gut microbiota imbalance, notably by enriching the beneficial gut bacteria, *Acidobacteria*, *Bacteroidetes*, and *Verrucomicrobia*, and reducing the harmful bacteria, *Proteobacteria* and *Cohaesibacter* (Kong et al., 2021). Selenium-rich polysaccharides extracted from *Cordyceps militaris* prevent HFD-induced hyperlipidemia in mice by reducing obesity-correlated gut bacteria, including *Dorea*, *Clostridium*, *Lactobacillus*, and *Ruminococcus*, and increasing the beneficial gut bacteria, *Akkermansia* (Yu et al., 2021). *Quinoa* polysaccharides derived from Inca food *Chenopodium quinoa*, ameliorate HFD-induced hyperlipidemia in rats by reducing *Firmicutes*/*Bacteroides* ratio, and the abundance of *Proteobacteria*, *Desulfovibrio*, and *Allobaculum* (Cao et al., 2020). Natural polysaccharides extracted from mushroom *Grifola frondose*, alleviate HFD-induced hyperlipidemia in rats by reversing HFD-induced intestinal flora dysbiosis, notably by increasing the abundance of *Helicobater*, *Barnesiella*, *Parasutterella*, and *Flavonifracter*, and decreasing the harmful bacteria, *Butyricicoccus* and *Turicibacter* (Li et al., 2019a). Oral administration of natural polysaccharides obtained from *Holothuria leucospilota* ameliorate hyperlipidemia in HFD-fed rats by increasing the SCFAs-producing gut microbiota (Yuan et al., 2019). Overall, natural polysaccharides can display probiotics-like activity in improving HFD-induced hyperlipidemia.

## HFD/Streptozotocin-Induced Type 2 Diabetes Mellitus

Diabetes mellitus is extremely common metabolic disease that affects the health of 460 million people in the world, and the number of diabetic patients will account for 9.9% of the global population by 2045 (Merino et al., 2019; Siehler et al., 2021). It seriously reduces quality of life, with many poor outcomes, including recurrent, longer hospital stays, and higher mortality rate (Nazzal et al., 2021). Approximately 90% of diabetic patients have T2DM, while T1DM represents 10% of all diabetes cases (Siehler et al., 2021). T2DM is an obesity-related disease, and increased dietary monounsaturated fat is linked with a higher risk of T2DM (Merino et al., 2019; Lingvay et al., 2021). Thus, combination of HFD and streptozotocin (STZ) is the most frequently used to establish alternative animal model of T2DM (Srinivasan et al., 2005). Currently, increasing evidence demonstrates that the gut microbiota can contribute to T2DM, and microbiota composition is altered in diabetic patients (Adeshirlarijaney and Gewirtz, 2020; Fan and Pedersen, 2021). In addition, the human microbiome contributes to the drug resistance of antidiabetic drug, acarbose (Balaich et al., 2021). Thus, targeting of gut microbiota is a defensive strategy against T2DM.

Natural polysaccharides can display prebiotics-like activities to improve T2DM by regulating the gut microbiota composition. *Cyclocarya paliurus* polysaccharides (CPP) alleviate T2DM symptoms in HFD/STZ-fed mice by increasing SCFAs contents, and promoting the SCFAs-producing gut species, such as *Ruminococcus* and *Anaerotruncus* (Yao et al., 2020). Natural polysaccharides derived from *Nigella sativa* seed, show antidiabetic effect in HFD/STZ-induced T2DM mice by increasing the abundance of intestinal microbiota *Bacteroides* and *Muribaculaceae* (Dong et al., 2020). *Grifola frondosa* polysaccharides (GFP) exhibit hypoglycemic and hypolipidemic effects in HFD/STZ-induced T2DM mouse model by reversing HFD/STZ-induced gut microbial dysbiosis, notably increasing *Alistipes*, and reducing *Streptococcus*, *Staphylococcus*, and *Enterococcus* (Guo et al., 2020). Tea polysaccharides extracted from *Camellia sinensis* L. possess hypolipidemic and hypoglycemic effect in HFD/STZ-induced T2DM rats by restoring the abundance of gut microbiota, such as *Lachnospira*, and *Victivallis*, and increasing the SCFAs contents (Li et al., 2020a). Polysaccharides isolated from *Cyclocarya paliurus* leaves attenuate diabetic symptoms in HFD/STZ-induced T2DM rats by increasing the SCFAs contents and the beneficial gut bacteria *Ruminococcaceae* (Li et al., 2021b). Natural polysaccharides from *Momordica charantia* ameliorate hyperglycemia, hyperlipidemia, and hyperinsulinemia in HFD/STZ-induced T2DM rats through increasing the SCFAs contents and the abundance of *Prevotella loescheii* and *Lactococcus laudensis* (Gao et al., 2018). Natural pumpkin polysaccharides extracted from popular vegetable *Cucurbita moschata*, show hypoglycemic effect in HFD/STZ-induced T2DM by increasing the biomarker, *Akkermansia*, and reducing *Erysipelotrichaceae* (Wu et al., 2021). In addition, pumpkin polysaccharides also increase the gut production of SCFAs in T2DM model (Liu et al., 2018). *Ganoderma lucidum* polysaccharides (GLP) display antidiabetic effects by restoring HFD/STZ-induced intestinal microbiota dysbiosis, notably by increasing *Blautia*, *Bacteroides*, *Dehalobacterium*, and *Parabacteroides*, and reducing the harmful gut bacteria, *Aerococcus*, *Corynebactrium*, *Ruminococcus*, and *Proteus* (Chen et al., 2020b). Natural polysaccharides from *Coix* seed, exhibit hypoglycemic activity in HFD/STZ-induced T2DM mouse model by reducing the *Firmicutes*/*Bacteroidetes* ratio, and increasing the contents of SCFAs (Xia et al., 2021). Glucomannans, as natural polysaccharides from *Dendrobium officinale*, *Aloe vera*, and *konjac*, ameliorates metabolic disorder of T2DM in HFD/STZ-fed rats through increasing the abundance of *Firmicutes*, and decreasing the abundance of *Bacteroidetes*, *Proteobacteria* (Chen et al., 2021a). Taken together, natural polysaccharides can prevent or treat T2DM by regulating gut microbiota composition and metabolites.

## HFD-Induced Non-Alcoholic Fatty Liver Disease

Non-alcoholic fatty liver disease remains a highly prevalent and largely underappreciated chronic liver disease that strongly affects about one-quarter of the global adults, and NAFLD is one of the most important risk factors of hepatocellular carcinoma (Foerster et al., 2021; Lazarus et al., 2021). The majority of patients suffering from NAFLD are obese, and NAFLD is considered as a further manifestation of metabolic syndrome (Tarantino and Finelli, 2015). Although the contributors of NAFLD are extremely complicated, emerging data demonstrates that HFD is a primary driver in the development of NAFLD (Jensen et al., 2018; Gao et al., 2020). Importantly, HFD induces adverse changes of intestinal microbiota composition in healthy young adults (Wan et al., 2019), and gut microbiota dysbiosis is highly associated with NAFLD (Leung et al., 2016). In this regard, HFD-induced gut microbiota disorder promotes the development of NAFLD by mediating the “gut-liver” axis (Le Roy et al., 2013). The communication between the gut and liver is through the biliary tract, portal vein, and systemic circulation (Tripathi et al., 2018). Currently, there is no approved drugs for the treatment of NAFLD, and several anti-NAFLD drugs are being undergoing various phases of recent clinical trials (Negi et al., 2022). However, natural polysaccharides extracted from plants exhibit great potential in alleviating NAFLD through manipulation of gut microbiota.

Gut microbe-derived microbial metabolites play pivotal roles in the development and progression of NAFLD, and these microbial metabolites include SCFAs, bile acids (BAs), choline metabolite trimethylamine (TMA), lithocholic acid (LCA), and succinate (Chu et al., 2019). SCFAs are the most abundant microbial metabolites that are generated from microbe fermentation of indigestible carbohydrates in gut (Dai et al., 2020). SCFAs are not only providers of nutrients and energy for intestinal epithelium, but also serve as signaling molecules to regulate intestinal lipogenesis and gluconeogenesis (Koh et al., 2016; Dai et al., 2020). Importantly, numerous human studies suggest that SCAFs are reduced in patients with NAFLD (Wang et al., 2016), and SCAFs supplementation is a therapeutic target for the prevention and treatment of HFD-associated NAFLD (Li et al., 2020c; Zhang et al., 2021a). Notably, natural polysaccharides play a major role in regulating gut microbiota to produce SCAFs (Liu et al., 2021a).

Astragalus polysaccharides (APS) are extracted from *Astragalus mongholicus* that attenuates NAFLD in HFD-fed mice, and mechanistically, APS enriches *Desulfovibrio* genus, especially *Desulfovibrio vulgaris* that is a generator of, SCFAs, acetic acid, and attenuates hepatic steatosis (Hong et al., 2021). Noni fruit polysaccharides are derived from *Morinda citrifolia* L. that alleviates NAFLD in HFD-fed mice through promoting SCFAs production, and reversing HFD-induced intestinal dysbiosis by improving gut microbiota diversity and composition (Yang et al., 2020b). Walnut green husk polysaccharides prevent obesity and NAFLD in HFD-fed rats by enhancing the SCFAs content and abundance of intestinal microbiota, including *Prevotellaceae*, *Allobaculum* (Wang et al., 2021). *Lycium barbarum* polysaccharides (LBP) are extracted from traditional Chinese herd and functional food, *Lycium barbarum* that combines with aerobic exercise to ameliorate NAFLD in HFD-fed rats by augmenting SCFAs contents and gut microbiota, *Bacteroidetes* (Gao et al., 2021b). In a randomized controlled trial, the potential prebiotics-like effect of LBP supplementation in treating NAFLD is being evaluated (Gao et al., 2021a). Natural polysaccharides, MDG-1, extracted from the roots of *Ophiopogon japonicus*, inhibits the progression of NAFLD in HFD-fed mice through restoring the gut microbiota balance by improving the abundance of SCFAs-producing the beneficial bacteria, *Butyricimonas* and *Roseburia* (Wang et al., 2019). Mussel polysaccharides, α-D-glucan (MPA) extracted from *Mytilus coruscus* protects NAFLD in HFD-fed rats, and mechanistically, MPA supplementation reverses HFD-inhibited microbial dysbiosis and SCFAs (Wu et al., 2019a). Soluble polysaccharides from *Laminaria japonica* attenuates NAFLD in HFD-fed mice by reducing the ratio of *Firmicutes*/*Bacteroidetes*, and promoting *Verrucomicrobia* and propionate-producing bacteria *Bacteroides* and *Akkermansia* (Zhang et al., 2021b).

Interestingly, the natural polysaccharides are not always safe for the treatment of NAFLD. For example, exopolysaccharides (EPS) derived from bacteria, *Lactobacillus rhamnosus* GG (LGG EPS) and *L. casei* BL23 (BL23 EPS), both ameliorate NAFLD and increase the acetate and propionate (SCFAs) in HFD-fed zebrafish, but BL23 EPS, not LGG EPS, induces liver inflammation and injury by intestinal microbiota dysbiosis (Zhang et al., 2019). In addition, *Cordyceps sinensis* polysaccharides (CSPs) exhibit obvious liver toxicity *via* aggravating liver steatosis and fibrosis in HFD-fed mice, and mechanistically, CSPs are digested by *Actinobacteria* and then excess *Actinobacteria* induces intestinal flora disorder and contributes to steatohepatitis (Chen et al., 2020a). Thus, using natural polysaccharides to treat NAFLD has a potential liver injury risk, and it is critical to select safe polysaccharides for treating NAFLD. Taken together, HFD induces gut microbiota dysbiosis that contributes to NAFLD, and intervention with natural polysaccharides can target the gut-liver axis, especially SCFAs, to alleviate or treat NAFLD. However, these findings on the efficacy of natural polysaccharides are consequence of studies in animal models, which is impossible completely mirror the human NAFLD. Thus, more clinical trials should be performed to further evaluate the efficacy of natural polysaccharides in NAFLD.

Sirtuins (SIRTs, SIRT1-7), ubiquitous deacetylases, are crucial metabolic regulators, and importantly, SIRTs are also emerging as the essential cause of NAFLD (Nassir and Ibdah, 2016; Palomer et al., 2021). SIRTs play master roles in a range of physiological functions, including fatty acid oxidation, mitochondrial oxidative metabolism, hepatic fat metabolism, and insulin secretion in hepatocytes (Nasrin et al., 2010; Laurent et al., 2013; Gupta et al., 2022). In NAFLD patients, SIRT1, SIRT2, SIRT3, SIRT5, and SIRT6 are dramatically down-regulated in livers (Wu et al., 2014; Ren et al., 2021), and plasma levels of SIRTs is highly associated with NAFLD (Mariani et al., 2015). In addition, low circulating levels of SIRT4 in obese NAFLD patients reduce oxidative capacity by reducing the mitochondrial ROS production in the liver, and in muscle (Tarantino et al., 2014). Therefore, SIRTs are the potential molecular targets for the treatment of NAFLD, as well as other metabolic diseases (Li et al., 2017). Notably, SIRTs can interact with the gut microbiota, and are linked with altered gut microbiota in NAFLD. For example, SIRT3 deficiency promotes HFD-induced NAFLD by impairing intestinal permeability through induction of the gut microbial dysbiosis (Chen et al., 2019). Thus, loss of SIRT3 interacts with the gut microbiota in the NAFLD progression. More importantly, natural polysaccharides regulate the activities of SIRTs, such as SIRT1 and SIRT3, in human diseases (Dimitrova-Shumkovska et al., 2020; Zhao et al., 2021b). However, the direct evidence that natural polysaccharides protect HFD-induced NAFLD through regulation of SIRTs-mediated gut microbiota is lacking. Therefore, SIRTs are novel research topics by which polysaccharides improve HFD-induced NAFLD by regulating gut microbiota.

## Metabolic Syndrome

Metabolic syndrome remains the most common non-communicable disease that is characterized by central obesity, insulin resistance, hyperlipidaemia, and hypertension (Bishehsari et al., 2020). MetS increases the risk of developing several chronic diseases, including T2DM, NAFLD, cardiovascular diseases, and cancer (Chen et al., 2021b; Kao and Huang, 2021). The contributors of MetS include genetic factors, and lifestyle, such as dietary over-consumption of HFD (Wutthi-In et al., 2020). Increasing studies show that the gut plays an important role in MetS, and the gut-centric theory in MetS emerged since 2007 (Fandriks, 2017; Dabke et al., 2019). Strong evidence for the important role of gut in MetS is the potent efficacy of weight-loss in gastrointestinal surgery (Hughes, 2014). Currently, intestinal microbiota is highly associated with MetS, and the gut microbiota dysbiosis results in obesity, and consequently MetS (Croci et al., 2021). Therefore, intervention of gut microbiota composition is an effective option for treating MetS patients (Wutthi-In et al., 2020; Guo et al., 2021b).

Natural polysaccharides have been demonstrated to alleviate or prevent MetS by regulating gut microbiota. Fucoidan, as sulfated polysaccharides from *Pearsonothuria graeffei*, alleviates MetS in HFD-fed mice by increasing abundances of *Actinobacteria* and *Bacteroidetes*, and decreasing the adverse bacteria, *Firmicutes* and *Proteobacteria* (Li et al., 2018). Flaxseed polysaccharides from *Linum usitatissimum*, prevent MetS in HFD-fed mice by increasing SCFAs contents and the beneficial bacteria, *Akkermansia*, *Bifidobacterium*, and decreasing the obesity-associated intestinal bacteria, *Oscillospira* and *Odoribacteraceae* (Yang et al., 2020a). Sulfated polysaccharides extracted from pacific abalone, improve MetS in HFD-fed mice by reversing HFD-reduced contents of SCFAs, and reducing ratio of *Firmicutes*/*Bacteroidetes* (Wu et al., 2020). Sulfated polysaccharides from *Isostichopus Badionotus*, prevent MetS in high fat and sucrose diet-fed mice by reducing the ratio of *Firmicutes*/*Bacteroidetes*, and the abundances of *Allobaculum*, *Lachnospiraceae*, and increasing abundances of *Barnesiella*, *Bacteroides*, and *Porphyromonadaceae* (Li et al., 2019b). Fuzhuan brick tea (FBT) polysaccharides extracted from *Camellia sinensis*, attenuate MetS in HFD-fed mice by restoring HFD-induced gut microbiota disorder, notably by reducing the harmful intestinal bacteria, *Coriobacteriaceae*, *Erysipelotrichaceae*, and *Streptococcaceae* (Chen et al., 2018). Natural polysaccharides extracted from *Flammulina velutipes*, attenuate MetS-related obesity, hyperlipidemia, and insulin resistance in HFD-fed mice by restoring HDF-induced gut microbiota dysbiosis and improving intestinal function (Zhao et al., 2021a). Sulfated polysaccharides from seafood *Undaria pinnatifida*, improve MetS in HFD-fed mice by reversing HFD-induced intestinal microbiota disorder, notably by increasing the beneficial bacteria, *Bacteroidetes*, and reducing the adverse bacteria, *Firmicutes* (Jiang et al., 2021). Therefore, supplementation of natural polysaccharides restores HFD-induced microbiota dysbiosis, and thereby improves MetS.

## Conclusion and Perspectives

**Figure 1:**
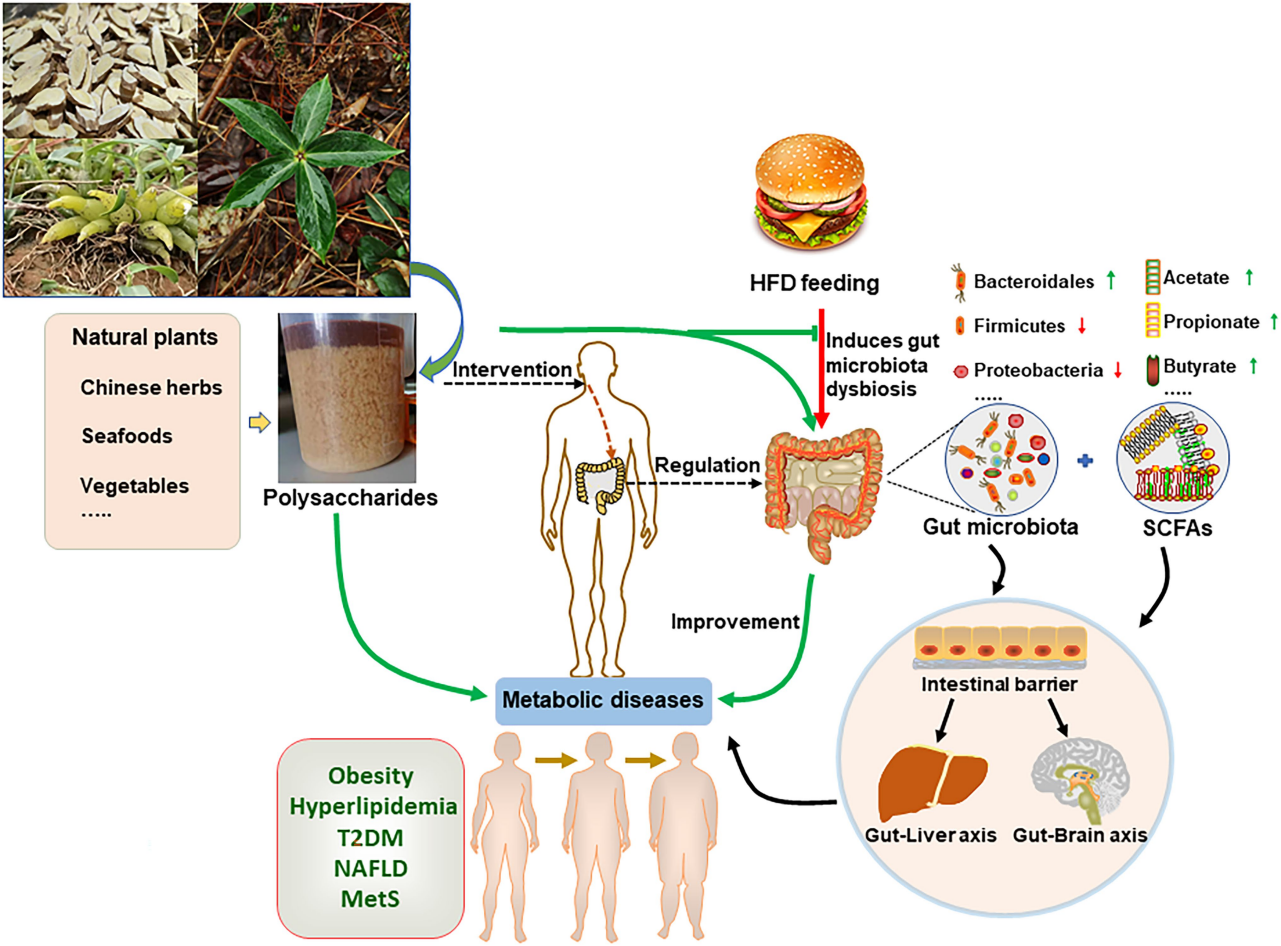
Natural polysaccharides improve high-fat diet (HFD)-induced metabolic diseases through regulating gut microbiota. Plants or animals-derived polysaccharides are fermented by gut microorganism, and produce metabolites, such as short-chain fatty acids (SCFAs). In turn, these metabolites regulate the intestinal microbiota composition. HFD induce gut microbiota dysbiosis that contributes to obesity-associated chronic metabolic diseases. In this regard, natural polysaccharides reverse HFD-induced metabolic diseases by regulating gut microbiota.

The composition of gut microbiota is highly linked with human health, and unhealthy lifestyles, in particular HFD, can induce chronic metabolic diseases by regulating gut microbiota. The plasticity of intestinal microbiota makes microbiome-targeted dietary intervention an important treatment for diseases. Natural polysaccharides are important components of human foods that are critical bidirectional regulators of gut microbiota. Gut microbiome ferments indigestible polysaccharides, and the fermented metabolites, in turn, affect intestinal microbiota composition and metabolites. Importantly, natural polysaccharides display probiotics-like activities in improving HFD-induced metabolic diseases (Figure 1; Table 1).

**Table 1:** The probiotic-like effect of polysaccharides in treating metabolic diseases.

Polysaccharides	Experimental models	Improvement of metabolic diseases	Regulation of gut microbiota	References
*Artemisia sphaerocephala* Krasch polysaccharides (200, 400, and 800 mg/kg)	HFD-fed KM mice (Obesity)	Reduce hepatic lipid metabolism, dyslipidemia, and metabolic endotoxaemia.	*Proteobacteria*, *AF12*, and *Helicobacter* ↓	Li et al., 2020b
*Dictyophora indusiata* polysaccharides (200, 400 mg/kg)	HFD-fed BALB/C mice (Obesity)	Reduce fat accumulation, adipocyte size, glucose levels, endotoxin, and inflammatory cytokines.	*Firmicutes*/*Bacteroidetes* ratio ↓	Kanwal et al., 2020
*Microalgae* polysaccharides (400 mg/kg)	HFD-fed C57BL/6 mice (Obesity)	Reduce glucose tolerance impairment, dyslipidemia, systemic inflammation, and fat deposition.	*Clostridia*, *Bacterioidia*, *Mollicutes* ↑ *Actinobacteria*, *Verrucomicrobia* ↓	Guo et al., 2021a
Modified apple polysaccharides (1,000 mg/kg)	HFD-fed C57BL/6J mice (Obesity)	Reduce body weight, fat index, lipid and glucose metabolism.	*Bacteroidetes*, *Bacteroides*, and *Lactobacillus* ↑ *Fusobacterium*↓	Li et al., 2020d
*Raphanus sativus* Polysaccharides (2, 4 mg/kg)	HFD-fed C57BL/6J mice (Obesity)	Reduce weight gain, and serum triglyceride, endotoxin; improve gut permeability.	*Verrucomicrobia* ↑ *Firmicutes*/*Bacteroidetes* ratio ↓	Do et al., 2021
*WuGuChong* polysaccharides (300 mg/kg)	HFD-fed C57BL/6 mice (Obesity)	Reduce liver steatosis, adipose hypertrophy, serum lipids, insulin resistance, and glucose tolerance.	*Firmicutes*/*Bacteroidetes* ratio, *Proteobacteria* ↓	Wang et al., 2020
*Hirsutella sinensis* polysaccharides (20 mg/kg)	HFD-fed C57BL/6J mice (Obesity)	Reduce systemic inflammation; improve insulin sensitivity, lipid metabolism, and gut integrity.	*Parabacteroides goldsteinii*. ↑	Wu et al., 2019b
*Brown seaweed* polysaccharides	HFD-fed BALB/c mice (Obesity)	Reduce weight gain, fat accumulation, lipid abnormality, and inflammation.	SCFAs contents, *Bacteroidales* ↑ *Clostridiales* ↓	Zheng et al., 2021
*Momordica charantia* Polysaccharides (50, 100, and 200 mg/kg)	HFD-fed SD rats (Obesity)	Improve the glycosphingolipids, glycerophospholipids, and amino acid metabolism.	*Actinobacteria*, *Coprococcus*, *Lactobacillus* ↑ *Proteobacteria*, and *Helicobacter* ↓	Wen et al., 2021
*Laminaria japonica* polysaccharides	HFD-fed BALB/c mice (Obesity)	Improve fat accumulation, lipids profile, body composition, and the morphology of the intestine.	*Bacteroidales*, *Rikenellaceae* ↑	Duan et al., 2019
*Pleurotus eryngii* polysaccharides	HFD-fed C57BL/6J mice (Obesity)	Reduce weight gain, serum cholesterol levels; improve lipid and total bile acids.	SCFAs-producing gut bacteria, *Anaerostipes*, *Clostridium* ↑	Nakahara et al., 2020
*Enteromorpha clathrate* polysaccharides (400 mg/kg)	HFD-fed C57BL/6J mice (Obesity)	Reduce the body weight and serum triacylglycerol and cholesterol levels.	SCFAs-producing gut bacterium, *Eubacterium xylanophilum* ↑	Wei et al., 2021
*Lycium barbarum* polysaccharides	HFD-fed ICR mice (Obesity)	Reduce serum cholesterol and triglycerides levels, the number and size of adipocytes.	SCFAs-producing gut bacteria, *Lacticigenium Lachnospiraceae*, and *Butyricicoccus* ↑	Yang et al., 2021b
*Polygonatum odoratum* Polysaccharides (400 mg/kg)	HFD-fed SD rats (Obesity)	Reduce weight gain, fat accumulation, adipocyte size, liver triglycerides, and liver cholesterol content.	SCFAs, isobutyric acid, butyric acid, valeric acid ↑ *Actinobacteria*, *Proteobacteria*, and *Sutterella* ↓	Wang et al., 2018b
*Ganoderma lucidum* Polysaccharides (100, 300 mg/kg)	HFD-fed C57BL/6J mice (Obesity)	Reduce fat accumulation hyperlipidemia, and inflammation; maintain intestinal barrier function.	SCFAs, acetate, and butyrate ↑	Sang et al., 2021
*Stichopus japonicus* Polysaccharides (300 mg/kg)	HFD-fed BALB/c mice (Obesity)	Reduce body weight, serum lipid, liver hypertrophy, insulin resistance, and inflammatory.	SCFAs contents, *Akkermansia* ↑ *Proteobacteria* ↓	Zhu et al., 2018
*Mulberry leaf* polysaccharides (200, 400, and 800 mg/kg)	HFD-fed C57BL/6J mice (Obesity)	Reduce body weight gain, and hepatic steatosis; improve lipid metabolism.	SCFAs contents ↑ *Firmicutes*/*Bacteroidetes* ratio ↓	Li et al., 2021c
*Ostrea rivularis* polysaccharides (125, 250, and 500 mg/kg)	HFD-fed zebrafish (Hyperlipidemia)	Reduce serum and hepatic lipid levels, and the hepatosomatic index, lipid droplets in hepatocytes.	*Acidobacteria*, *Bacteroidetes*, and *Verrucomicrobia* ↑ *Proteobacteria*, *Cohaesibacter* ↓	Kong et al., 2021
Selenium-Rich *Cordyceps militaris* polysaccharides (50, 100, and 200 mg/kg)	HFD-fed C57BL/6J mice (Hyperlipidemia)	Reduce the body weight, fat content, serum lipid, lipid gene expression, appetite hormone, and inflammation.	*Akkermansia* ↑ *Dorea*, *Clostridium*, *Lactobacillus*, and *Ruminococcus* ↓	Yu et al., 2021
*Chenopodium quinoa* polysaccharides (300, 600 mg/kg)	HFD-fed SD rats (Hyperlipidemia)	Reduce serum total triglyceride (TG), cholesterol, malondialdehyde (MDA), and total glutamic pyruvic transaminase.	*Firmicutes*/*Bacteroides* ratio ↓ *Proteobacteria*, *Desulfovibrio*, and *Allobaculum* ↓	Cao et al., 2020
*Grifola frondose* polysaccharides (100, 400 mg/kg)	HFD-fed Wistar rats (Hyperlipidemia)	Reduce serum total triglyceride levels, total cholesterol, free fatty acids, and hepatic lipid accumulation and steatosis.	*Helicobater*, *Barnesiella*, *Parasutterella*, *Flavonifracter* ↑ *Butyricicoccus* and *Turicibacter* ↓	Li et al., 2019a
*Holothuria Leucospilota* Polysaccharides (100, 200 mg/kg)	HFD-fed Wistar rats (Hyperlipidemia)	Reduce serum lipid levels, liver histological abnormalities, lipogenesis-related hormones, and inflammatory.	SCFAs contents ↑	Yuan et al., 2019
*Cyclocarya paliurus* polysaccharides (400 mg/kg)	HFD/STZ-fed SD rats (T2DM)	Reduce blood glucose levels; improve glucose tolerance, and serum lipid parameters.	SCFAs contents, SCFAs-producing gut microbiota, *Ruminococcus*, *Anaerotruncus* ↑	Yao et al., 2020
*Nigella sativa seed* polysaccharides (35, 70, and 140 mg/kg)	HFD/STZ-fed KM mice(T2DM)	Reduce fasting blood glucose, glycosylated serum protein, cholesterol, triglycerides, malondialdehyde, and inflammatory, and improve insulin resistance.	*Bacteroides*, *Muribaculaceae* ↑	Dong et al., 2020
*Grifola frondosa* polysaccharides (300, 900 mg/kg)	HFD/STZ-fed KM mice(T2DM)	Reduced fasting blood glucose (FBG), glucose tolerance, cholesterol, triglyceride, and hepatic free fatty acids.	*Alistipes* ↑ *Streptococcus*, *Staphylococcus*, and *Enterococcus* ↓	Guo et al., 2020
*Camellia sinensis* polysaccharides (100, 200, and 400 mg/kg)	HFD/STZ-fed Wistar rats (T2DM)	Reduce fasting blood glucose, and total cholesterol and triglyceride levels, and free fatty acid.	*Lachnospira*, and *Victivallis*, SCFAs contents ↑	Li et al., 2020a
*Cyclocarya paliurus* polysaccharides (200, 300, and 400 mg/kg)	HFD/STZ-fed Wistar rats (T2DM)	Reduce fasting blood glucose, inflammation, and serum hormones; improve insulin sensitivity.	SCFAs contents, *Ruminococcaceae* ↑	Li et al., 2021b
*Momordica charantia* polysaccharides (50, 100, and 200 mg/kg)	HFD/STZ-fed Wistar rats (T2DM)	Reduce fasting blood glucose, insulin levels, serum lipids, hyperglycemia, hyperlipidemia and oxidative stress.	SCFAs contents, *Prevotella loescheii*, *Lactococcus laudensis* ↑	Gao et al., 2018
*Pumpkin* polysaccharides (100, 200 mg/kg)	HFD/STZ-fed C57BL/6J mice (T2DM)	Reduce fasting blood glucose, insulin resistance, and blood lipid levels.	*Akkermansia* ↑ *Erysipelotrichaceae* ↓	Wu et al., 2021
*Pumpkin* polysaccharides (100, 200 mg/kg)	HFD/STZ-fed Wistar rats (T2DM)	Improve insulin tolerance, and reduce the levels of serum glucose and total cholesterol.	SCFAs contents ↑	Liu et al., 2018
*Ganoderma lucidum* polysaccharides (400 mg/kg)	HFD/STZ-fed SD rats (T2DM)	Reduce fasting blood glucose, and insulin, total cholesterol, and systematic inflammation; and improve anti-oxidant ability.	*Blautia*, *Bacteroides*, *Dehalobacterium*, *Parabacteroides* ↑ *Aerococcus*, *Corynebactrium*, *Ruminococcus*, and *Proteus* ↓	Chen et al., 2020b
*Coix* seed polysaccharides (175, 350 mg/kg)	HFD/STZ-fed C57BL/6J mice (T2DM)	Reduce fasting blood glucose, body weight, serum lipid parameters; and improve glucose tolerance	SCFAs contents ↑ *Firmicutes*/*Bacteroidetes* ratio ↓	Xia et al., 2021
Glucomannans (160 mg/kg)	HFD/STZ-fed Wistar rats (T2DM)	Reduce fasting blood glucose.	*Firmicutes* ↑ *Bacteroidetes*, *Proteobacteria* ↓	Chen et al., 2021a
*Astragalus mongholicus* polysaccharides	HFD-fed C57BL/6J mice (NAFLD)	Reduce body weight, fat index, liver triglycerides, hepatic steatosis, and pro-inflammatory cytokines.	*Desulfovibrio* genus, especially *D. vulgaris*↑	Hong et al., 2021
Noni fruit polysaccharides (100 mg/kg)	HFD-fed SD rats (NAFLD)	Reduce body weight gain, and improve lipid metabolism, and hepatic oxidative stress, inflammation.	SCFAs contents ↑ gut microbiota diversity and composition ↑	Yang et al., 2020b
*Walnut green husk* polysaccharides (600 mg/kg)	HFD-fed SD rats (NAFLD)	Reduce weight gain, inflammation, and improve oxidative stress, lipid metabolism, and colonic tissue injury.	SCFAs content, *Prevotellaceae*, *Allobaculum* ↑	Wang et al., 2021
*Lycium barbarum* polysaccharides (50 mg/kg)	HFD-fed SD rats (NAFLD)	Reduce hepatic inflammation, and improve intestinal barrier, insulin resistance, glucose tolerance, and lipid metabolic indices.	SCFAs contents, *Bacteroidetes* ↑	Gao et al., 2021b
*Ophiopogon japonicus* polysaccharides	HFD-fed C57BL/6J mice (NAFLD)	Improve hepatic lipid metabolism, liver injury, serum and intestinal inflammatory.	SCFAs-producing bacteria, *Butyricimonas*, *Roseburia* ↑	Wang et al., 2019
*Mussel* polysaccharides	HFD-fed SD rats (NAFLD)	Reduce blood lipid levels, hepatic triglyceride and lipid accumulation, and AST, ALT.	SCFAs contents ↑	Wu et al., 2019a
*Laminaria japonica* polysaccharides	HFD-fed C57BL/6 mice (NAFLD)	Reduce serum triglycerides, glucose, cholesterol, liver steatosis and hepatocellular ballooning.	*Verrucomicrobia*, *Bacteroides*, and *Akkermansia* ↑ *Firmicutes*/*Bacteroidetes* ratio ↓	Zhang et al., 2021b
*Pearsonothuria graeffei* polysaccharides (20, 80 mg/kg)	HFD-fed C57BL/6J mice (MetS)	Reduce weight gains, serum inflammatory cytokines, macrophages infiltrating; and improve hyperlipidemia, and liver steatosis.	*Actinobacteria*, *Bacteroidetes* ↑ *Firmicutes*, *Proteobacteria* ↓	Li et al., 2018
*Flaxseed* polysaccharides	HFD-fed C57BL/6 J mice (MetS)	Reduce serum fasting glucose, total triglyceride, and total cholesterol levels.	SCFAs contents, *Akkermansia*, *Bifidobacterium* ↑ *Oscillospira*, *Odoribacteraceae* ↓	Yang et al., 2020a
*Pacific abalone* polysaccharides	HFD-fed BALB/c mice (MetS)	Reduce weight gain, fat accumulation; and improve lipid metabolism.	SCFAs contents ↑ *Firmicutes*/*Bacteroidetes* ratio ↓	Wu et al., 2020
*Isostichopus Badionotus* polysaccharides (20, 40 mg/kg)	HFD-fed C57BL/6J mice (MetS)	Improve obesity, hyperglycemia, hyperlipidemia, liver steatosis, inflammation, and adipocyte hypertrophy.	*Barnesiella*, *Bacteroides*, *Porphyromonadaceae* ↑ *Firmicutes*/*Bacteroidetes* ratio, *Allobaculum*, *Lachnospiraceae* ↓	Li et al., 2019b
Fuzhuan brick tea polysaccharides (200, 400, and 800 mg/kg)	HFD-fed C57BL/6 mice (MetS)	Reduce body weight gain, liver weight, and hepatic lipid deposition.	*Coriobacteriaceae*, *Erysipelotrichaceae* ↑ *Streptococcaceae* ↓	Chen et al., 2018
*Flammulina velutipes* polysaccharides	HFD-fed C57BL/6J mice (MetS)	Improve obesity, hyperlipidemia and insulin resistance.	*Bifidobacteriaceae*, *Lactobacillaceae* *Firmicutes*/*Bacteroidetes* ratio ↓	Zhao et al., 2021a
*Undaria pinnatifida* polysaccharides (500 mg/kg)	HFD-fed BALB/c mice (MetS)	Reduce weight gain, fat accumulation and improve metabolic disorders.	*Bacteroidetes* ↑ *Firmicutes* ↓	Jiang et al., 2021

Nevertheless, a few limitations and challenges exist to use natural polysaccharides for the treatment of high-fat-associated metabolic diseases through gut microbiota. First, a comprehensive understanding of the interaction between gut microbiota and natural polysaccharides needs further studies. Second, because most of researches are in animal, it is critical to perform the clinical trials that examine the polysaccharides-microbiome-diseases interaction, and realization of clinical translation. Third, the adverse effects of polysaccharides, including hepatotoxicity, should be evaluated prior to clinical use.

## Author Contributions

C-YS and Z-LZ drafted the manuscript. C-YS and B-WL revised the manuscript. C-WC and DL approved the final manuscript.

## Funding

This study is supported by the grants from the Major Science and Technology Project of Anhui Province (Nos: 202003c08020004 and 202103b06020004).

## Conflict of Interest

The authors declare that the research was conducted in the absence of any commercial or financial relationships that could be construed as a potential conflict of interest.

## Publisher’s Note

All claims expressed in this article are solely those of the authors and do not necessarily represent those of their affiliated organizations, or those of the publisher, the editors and the reviewers. Any product that may be evaluated in this article, or claim that may be made by its manufacturer, is not guaranteed or endorsed by the publisher.
